# Six potential biomarkers for bladder cancer: key proteins in cell-cycle division and apoptosis pathways

**DOI:** 10.1186/s43046-022-00153-0

**Published:** 2022-12-19

**Authors:** Güldal Inal Gültekin, Özlem Timirci Kahraman, Murat Işbilen, Saliha Durmuş, Tunahan Çakir, İlhan Yaylim, Turgay Isbir

**Affiliations:** 1 Department of Physiology, Faculty of Medicine, Istanbul Okan University, Tepeören Campus, Tuzla, Istanbul, Turkey; 2 Department of Molecular Medicine, Istanbul University, Aziz Sancar Experimental Research Institute, Çapa, Istanbul, Turkey; 3 Department of Biostatistics and Bioinformatics, Acibadem Mehmet Ali Aydinlar University, Istanbul, Turkey; 4 Department of Bioengineering, Faculty of Engineering, Gebze Technical University, Kocaeli, Turkey; 5 Department of Molecular Medicine, Faculty of Medicine, Yeditepe University, Kayışdağı, Istanbul, Turkey

**Keywords:** Cell division, Differentially expressed genes, Integrated bioinformatics, LGALS3, AURKB, Intrinsic apoptosis

## Abstract

**Background:**

The bladder cancer (BC) pathology is caused by both exogenous environmental and endogenous molecular factors. Several genes have been implicated, but the molecular pathogenesis of BC and its subtypes remains debatable. The bioinformatic analysis evaluates high numbers of proteins in a single study, increasing the opportunity to identify possible biomarkers for disorders.

**Methods:**

The aim of this study is to identify biomarkers for the identification of BC using several bioinformatic analytical tools and methods. BC and normal samples were compared for each probeset with *T* test in GSE13507 and GSE37817 datasets, and statistical probesets were verified with GSE52519 and E-MTAB-1940 datasets. Differential gene expression, hierarchical clustering, gene ontology enrichment analysis, and heuristic online phenotype prediction algorithm methods were utilized. Statistically significant proteins were assessed in the Human Protein Atlas database. GSE13507 (6271 probesets) and GSE37817 (3267 probesets) data were significant after the extraction of probesets without gene annotation information. Common probesets in both datasets (2888) were further narrowed by analyzing the first 100 upregulated and downregulated probesets in BC samples.

**Results:**

Among the total 400 probesets, 68 were significant for both datasets with similar fold-change values (Pearson *r*: 0.995). Protein-protein interaction networks demonstrated strong interactions between CCNB1, BUB1B, and AURKB. The HPA database revealed similar protein expression levels for CKAP2L, AURKB, APIP, and LGALS3 both for BC and control samples.

**Conclusion:**

This study disclosed six candidate biomarkers for the early diagnosis of BC. It is suggested that these candidate proteins be investigated in a wet lab to identify their functions in BC pathology and possible treatment approaches.

**Supplementary Information:**

The online version contains supplementary material available at 10.1186/s43046-022-00153-0.

## Background

Bladder cancer (BC) is a widespread malignant tumor of the genitourinary system. It has a high mortality and recurrence risk, thus posing a significant health risk worldwide. The incidence rate of BC is proportional to the developmental stage of the region, but also depends on the developmental level of the population. In both situations, its incidence rises as the developmental stage increases [1].

The prevalence of BC is multidimensional; furthermore, the causative processes in its occurrence and progression are complex. Its pathology is caused by both exogenous environmental and endogenous molecular factors. Smoking and tobacco usage is one of the leading factors [2], which can be preventable. However, BC is still highly prevalent in men and women in all continents [1]. Last but the least, the Human Developmental Index (HDI) levels have been clearly implicated in several databases to be proportional to BC incidence [1, 3, 4]. Several genes have been implicated in the pathogenesis of BC [5, 6]. The treatment options for BC include conventional chemotherapy and immune checkpoint inhibitors, but fail to treat every individual [7]. Hence, personalized approaches and precise biomarker discoveries remain important.

Currently, the most common source of large-scale molecular data is transcriptome data for malignancies. High-throughput approaches such as microarray technology generate a large amount of genomic and expression data which are publicly available through various online repositories. The generation of big data necessitates the employment of high-throughput computer approaches and several methodologies can be utilized for statistical analysis. Bioinformatics, which is the intersection of biology, information science, and computation, was once an emerging component of cancer research [8], but now is one of the most common initial approaches prior to wet lab studies.

## Methods

The aim of this research is to identify biomarkers for the early diagnosis and treatment of BC by using a variety of bioinformatic analytical tools and methods.

### BC gene expression data sources

Four different datasets were downloaded from the gene expression omnibus (GEO) database (geneontology.org), including GSE13507 [Illumina] [9], GSE37817 [Illumina] [10], GSE21519 [Illumina] [11], and E-MTAB-1940 [Affymetrix] [12]. The latter two were used for validation studies. The datasets were downloaded from the ArrayExpress database in a normalized arrangement, and Affymetrix gene IDs were obtained accordingly (ebi.ac.uk/arrayexpress/).

### Data processing and statistical tests

R 3.4.0 programming language was utilized for the statistical tests [13]. Student’s *T* test was used to compare gene expression levels in different groups.

### Experimental design

BC and normal samples were compared for each probeset by *T* test in the GSE13507 and GSE37817 datasets with Venn diagrams using the online tool Venny 2.1 [14], and volcano plots were generated. The analysis was restricted to the first 100 upregulated and downregulated probesets. The median values of common probesets in the BC and normal samples were calculated. Then, “Pearson r” values were calculated for the gene expression values. With this approach, tumor and normal “*r* values” were obtained for all samples. Hierarchical clustering analysis was performed with Cluster 3.0 using Euclidean distance and complete linkage parameters [15]. The Heuristic Online Phenotype Prediction (HOPP) algorithm was applied as previously reported [16].

### Functional enrichment analysis

Gene ontology (GO) enrichment analyses were performed using the PANTHER Classification Systems database [17]. Gene sets at *p*<0.05 were considered to be significant.

### Candidate hub gene identification

A network analysis was performed using the Search Tool for the Retrieval of Interaction Genes 10.5 (STRING) database [18]. Protein-protein interaction (PPI) networks for differentially expressed genes (DEGs) were built on high confidence (0.700) interaction scores.

## Results

**Fig. 1:**
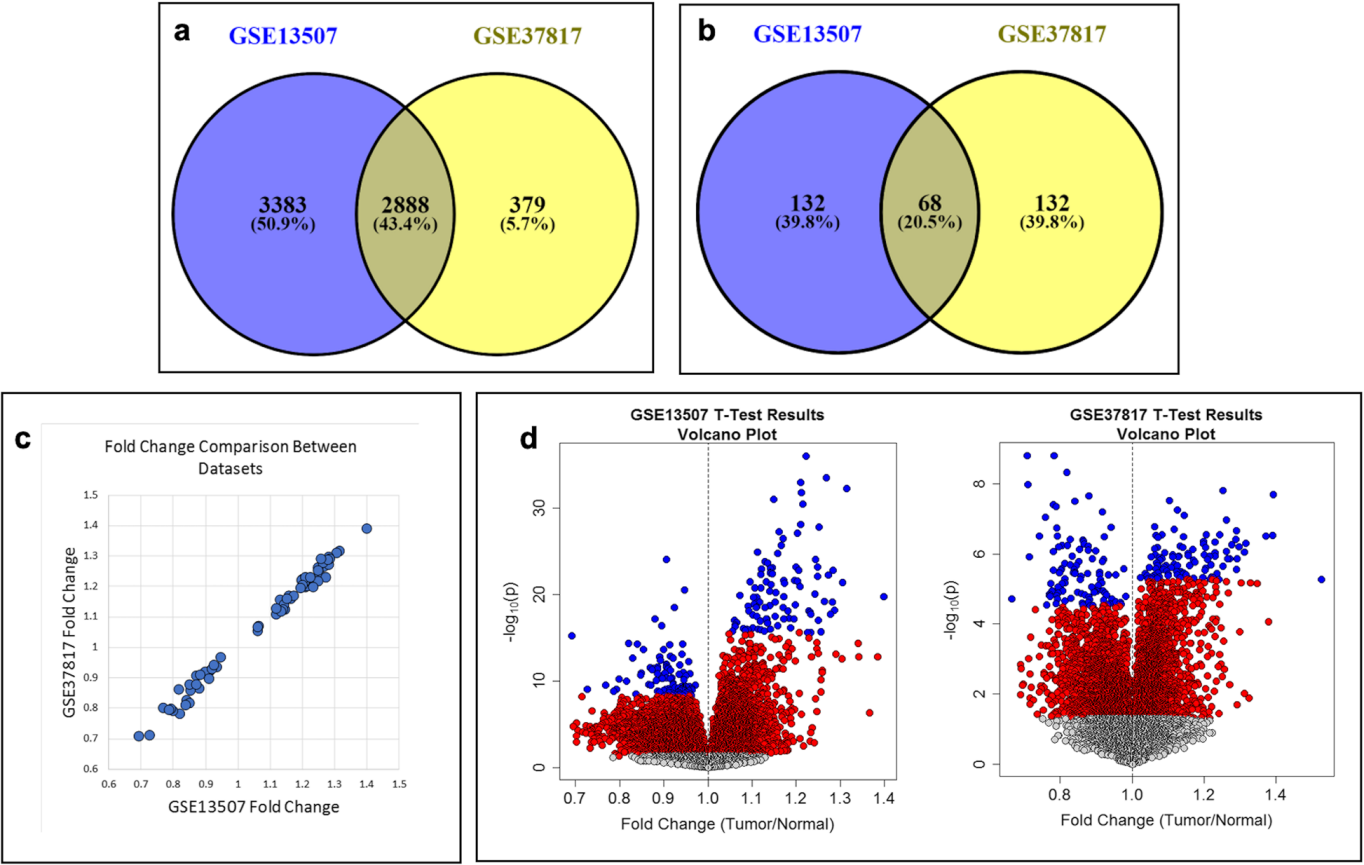
Common probesets between GSE13507 and GSE37817 datasets are determined using Venny diagrams **a** without restriction [common 2888 probesets, 34.4%] and **b** with restriction to the first 100 upregulated and downregulated in both datasets [common 68 probesets, 20.5%], **c** mutual probesets expressed similar fold-change values in the same direction (Pearson r: 0.995), **d** all probesets in both datasets were represented by volcano plots where each point represent a single probeset with fold change values on the *x* axis and logarithmic *t* test *p* value on the *y* axis [red and blue (first 100) dots indicating significant probesets, gray dots indicating non-significant probesets]

Following the subtraction of probesets lacking gene annotation information, the comparison provided 6271 and 3267 significant probesets in GSE13507 and GSE37817 datasets, respectively (Fig. 1a). These two datasets showed significant overlapping for 2888 probesets (43.4%). To narrow the significant probesets, the first 100 upregulated and downregulated probesets in the tumor were selected with respect to their order of significance. For this purpose, a total of 200 probesets were compared from both datasets (GSE13507, GSE37817). Among these 400 probesets, 68 (20.5%) were significantly overlapping (Fig. 1b) and these mutual probesets showed similar fold-change values in the same direction (Pearson r: 0.995) (Fig. 1c; Table S1). All probesets were visualized in Volcano plots (Fig. 1d).

### GO enrichment analysis

**Fig. 2:**
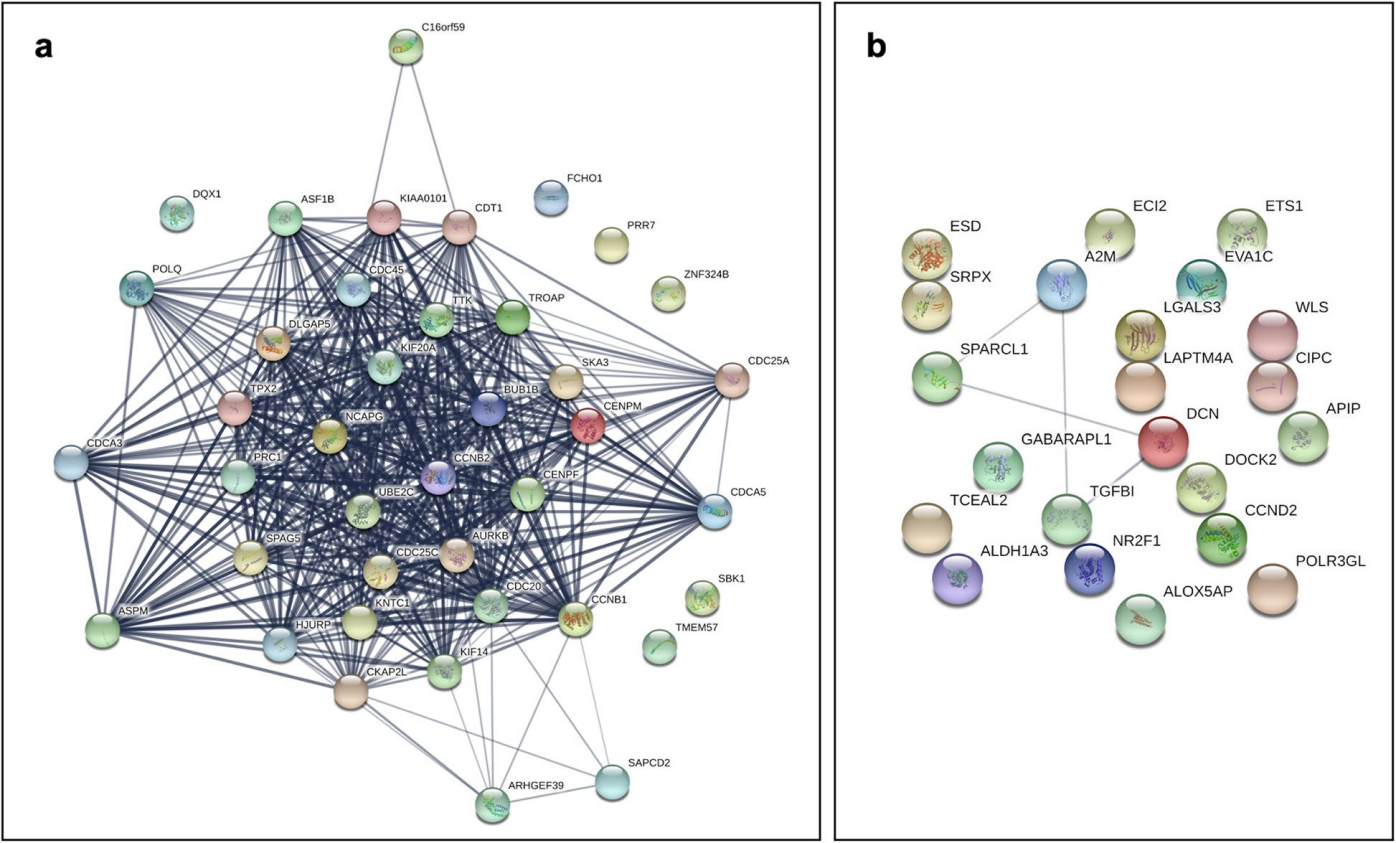
The protein-protein interaction (PPI) analysis reveals **a** significant core networks between mutually upregulated differentially expressed genes (DEGs), but **b** nearly no connections between downregulated DEGs were observed

Among the 68 mutually significant probesets, a GO enrichment analysis was performed for the 42 upregulated (Fig. 2a) and 26 downregulated genes (Fig. 2b). The genes were enriched for biological processes, cellular components, and molecular functions. Some statistically significant pathways included, but were not limited to, GO:0051301: Cell division (fold enrichment: 23.72, *p*=7.1E−22); GO:0000280: Nuclear division (fold change: 28.69, *p*= 1.4E−14); GO:0140014: Mitotic nuclear division (fold change: 46.42, *p*= 2.2E−13); GO:0000070: Mitotic sister chromatid segregation (fold change: 52.08, *p*=4.2E−11); GO:1902099: Regulation of metaphase/anaphase transition of cell cycle (fold change: 78.35, *p*=4.2E−08); GO:0031577: Spindle checkpoint (fold change: 91.49, *p*= 1.1E−03); GO:0006260: DNA replication (fold change: 14.68, *p*= 2.8E−02). The PPI analysis indicates a strong interaction between all 42 upregulated proteins. Particularly, strong interactions between CCNB1, BUB1B, AURKB, CCNB2, CDC20, CDCA5, CENPF, and KNTC1 proteins were noticeable.

### Validation

**Fig. 3:**
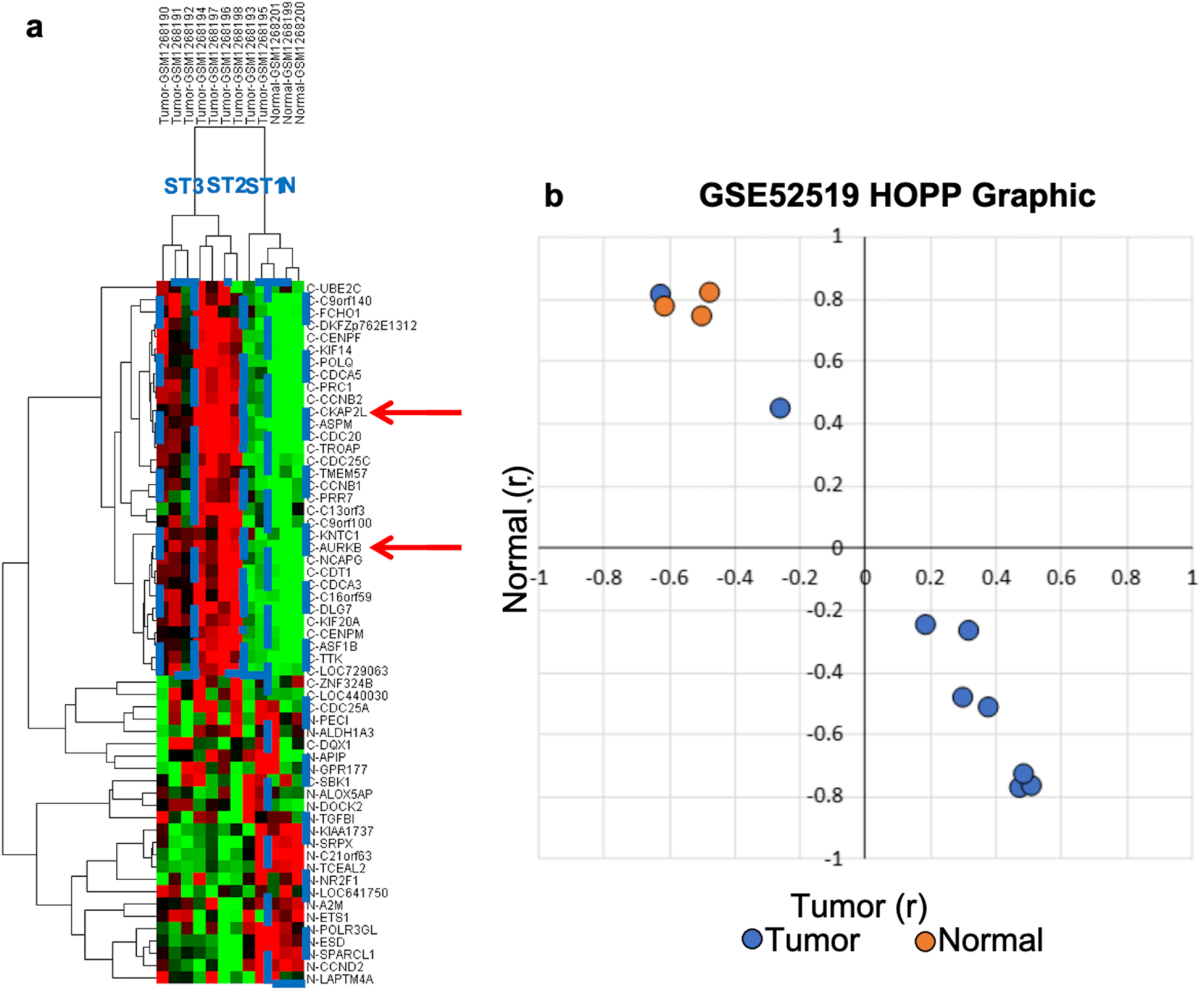
The first validation study conducted with the GSE52519 dataset **a** revealed four clusters for the 57 mutual probesets, the rightmost group in the heat map represents the normal samples (*N*), two BC samples were located in the same cluster (ST1), four BC samples were clustered in the third group (ST2), and the fourth cluster included three BC samples that clustered separately (ST3); this validation included the *CKAP2L* and *AURKB* genes [red arrows] that were upregulated in ST2 and ST3 [BC and control samples are represented on the vertical axis, probes (genes) on the horizontal axis; red, black, and green colors represent high, medium, and low gene expression levels, respectively]. **b** The Heuristic Online Phenotype Prediction (HOPP), a gene expression analysis algorithm that employs PEARSON (*r*) correlations, grouped both (normal and BC) phenotypes and predicted two BC samples along with the normal sample

For the mutual 68 genes, two validation studies were performed using the GSE52519 and E-MTAB-1940 datasets. Out of the 68 probesets, 11 were missing in the GSE52519 dataset; hence, the validation was performed with 57 probesets (excluded probesets: ILMN_1792494, ILMN_1768291, ILMN_1808347, ILMN_1733950, ILMN_1663332, ILMN_1745594, ILMN_1747118, ILMN_1750347, ILMN_171251667, ILMN_1712574). The GSE52519 dataset included 9 BC samples and 3 healthy bladder samples. Normal samples were distinguished from BC samples with a hierarchical clustering analysis (Fig. 3a). Where BC samples were observed to form three clusters, subtypes 1, 2, and 3 (ST1, ST2, ST3). Two tumor samples showed closer proximity to normal samples, and this was observed with the HOPP (Fig. 3b).

The second validation performed with the E-MTAB-1940 dataset was a 96 Affymetrix platform. Hence, the Affymetrix probesets corresponding to the genes of interest were determined, and out of the 68 genes, 52 were identified. The hierarchical clustering analysis performed with these probesets showed some separation between normal and tumor samples. Although, many tumor samples were clustered in the same group with normal samples, and one normal sample was clustered with tumor samples (Fig. S1).

### Protein and tumor database analysis

The investigation of the common 68 proteins in The Human Protein Atlas database ([19]; proteinatlas.org) revealed a similar association between tumor and normal tissues at the protein level for CKAP2L, AURKB, CDC25A, APIP, and LGALS3.

## Discussion

In this study, we performed bioinformatic analysis using microarray analysis to identify biomarkers related to BC which hold the potential to substantially sort between protein patterns for BC and normal tissue. Four datasets were used herein to categorize DEGs and validate results. The fact that the common probes were excessively high, we opted to select the first 100 upregulated and downregulated probes in tumor samples. With this approach, the high number of significant probesets (2888), which is an indication that the probability of false positives may be low, was narrowed to 68 common significant probesets. The comparable fold-change values of the shared probesets in both datasets behaved in the same manner. That is, the common proteins were upregulated and downregulated in both datasets. This is indicative that the effect of these common probesets on the formation of tumorigenesis can be determined independently in different experiment settings, thus possessing strong differentiating properties between cancer and healthy tissue.

### GEO and STRING analysis

The GO enrichment analysis conducted with the 68 common probes demonstrates that most of these genes have functions related to cell division and mitosis; additionally, they were mostly found to encode proteins found in the “cell cycle-related pathways.” These included “regulation of cell cycle checkpoints” and “separation of sister chromatids,” which are key pathways of cellular and nuclear division, and are proposed to lead to cancer development [20]. The lack of commonly downregulated genes in BC samples indicates that these genes may have false-positive findings. Nevertheless, GEO data and STRING analysis results suggest that the trigger for BC development may be by accelerating biological mechanisms and pathways rather than inhibiting them. This could contribute to the selection of drugs and treatment approaches that suppress the accelerated pathways.

Specifically, the interaction between eight DEGs (CCNB1, CCNB2, CDC20, CDCA5, CENPF, KNTC1, BUB1B, and AURKB) was particularly apparent. CCNB1 has previously been reported in several studies on BC [21–23]. It encodes a key regulatory protein involved in the replication of nuclear matter by creating a complex with *CDC2* (cell division cycle) also known as *p34*. Together, they control the mitosis at the G2/M-specific checkpoint [24]. Although CDC2 was not detected in this study, other regulatory genes responsible for the cell division cycle were enriched in the GEO and STRING analysis. This is likely to imbalance in the mitotic activity in BC samples. The *CCNB1* gene is located on chromosome 5q13.2 and is composed of 9 exons; it encodes a 2029 bp mRNA and its longest transcript encodes a protein of 433 aa. Some SNPs on this gene have been previously reported in relation to cancer development; however, these SNPs have not been assessed in BC yet.

The spindle checkpoint kinase BUB1B (BUB1 mitotic checkpoint serine/threonine kinase beta) is involved in mitotic checkpoint and has recently been reported in a study as a hub candidate gene for BC [24]. It is thought to be localized to the kinetochore and delays the anaphase-promoting complex/cyclosome, enabling chromosomes to properly segregate [25]. Located on chromosome 15q15.1, it encodes an mRNA of 3669 bp and a protein of 1050 aa, and its impaired activity has been implicated in the formation of breast cancer [26].

Furthermore, similar oncogenes, including CCNB1, CDC20, and AURKA from the aurora kinase group were reported as hub genes by Zhang et al. [27], recently. Our study also supports their Go and KEGG enrichment analyses of DEGs, which implies the importance of mitotic checkpoints.

### Validations studies

Two validation studies were conducted. The first one showed clear differentiation between tumor and normal samples. Almost none of the upregulated genes in tumor samples had high expression in normal samples. However, several upregulated genes in normal samples were also upregulated in some of the BC samples. Thus, genes with increased expression in the BC showed an enhanced discrimination power. This initial validation clustered the probes into four distinct groups. Two BC samples (ST1) were clustered with healthy tissues (N) and did not express the upregulated genes in BC, but rather expressed genes that were downregulated in tumor samples. The ST2 cluster containing 4 tumor samples showed increased expression in genes that were upregulated in the tumors. They behaved as expected from tumor samples. The final cluster (ST3) included 3 BC samples and expressed slightly less the upregulated genes in tumor samples. This indicates that based on the common 57 gene expressions, BC samples revealed subgroups that are comparable to or distinct from healthy tissue samples. Due to the fact that the datasets lacked information on tumor subtypes, a clustering of probes based on BC subtypes could not be implemented in this study. Nevertheless, provided that the dataset includes the BC subtype information, this can be accomplished and might offer alternative approaches and specific genes for personalized treatments.

The second validation dataset, although acquired from a different platform, was also capable of distinguishing between subtypes based on the common 52 genes out of 68, but to a lower extent. This dataset was composed of 86 samples with 4 normal samples and separated the BC samples into 3 subtypes. The distinction was not as apparent as it was in the first validation, due to the fact that there were multiple probesets corresponding to the same gene. Moreover, some probesets had no contribution to the separation, but nevertheless, a separation between BC subtypes was still noticeable.

Thus, based on the validation studies, the 68 featured proteins have been produced in two different platforms and distinguished not just BC from normal tissue but also differentiated between subtypes of BC.

### Protein database

Protein database investigations led to the prominence of four of these 68 proteins (CKAP2L, AURKB, APIP, LGALS3) which were found consistent with the results of statistical protein levels presented herein. CKAP2L (cytoskeleton-associated protein 2-like) is involved in spindle organization and cell cycle progression from prometaphase to telophase [28]. AURKB (aurora kinase B), a member of the kinase family, is thought to have a role in the control of chromosomal alignment and segregation during mitosis by interacting with microtubules. A group of small-molecule inhibitors of AURKB, with ongoing or completed Phase I and II trials, have recently been proposed as potential drugs for cancer treatment [29]. Given that the expression levels of CKAP2L and AURKB statistically increased in BC samples, this could be a promising approach to investigate. APIP (APAF-1 interacting protein) is a protein found mostly in the cytosol and interacts with Apaf-1 (apoptotic protease activating factor-1), which holds a central role in the initiation to form the apoptosome complex and downstream pathway to intrinsic apoptosis [30]. APIP is thought to block the intrinsic mitochondrial apoptosis pathway via two routes, one Apaf-1-dependent [31] and the other Apaf-1-independent [32]. The downregulation of APIP expression in BC tissue samples could, however, be indicative of the absence of a regulatory protein. Similarly, mRNA and protein expressions of APIP were reported downregulated in non-small cell lung carcinoma [33]. LGALS3 (galectin 3), a member of the galectin family of carbohydrate-binding proteins, has previously been reported to induce apoptosis in human breast cancer cell lines through TRAIL signals that were dependent on increased PTEN activation and decreased PI3K/AKT survival pathway [34]. Downregulation of LGALS3 in the BC tissue most likely intervenes with TRAIL-induced apoptotic pathways. However, these findings contradict Oka et al. [35], who reported that overexpression of LGALS3 protects J82 human bladder cancer cells against TRAIL-induced apoptosis [35]. This contradiction can most likely be explained by the diverse apoptotic molecular pathways in different cell lines [34, 35].

BC is one of the most studied cancer types in the bioinformatic analysis due to its prevalence in humanity [27, 36, 37]. However, the use of different datasets as well as various types of statistical approaches, which improve constantly diverse biomarkers is constantly being predicted for BC [27, 36, 37], thus underlying the multiple factorial nature of BC and increasing the value of bioinformatic studies.

## Conclusions

Considering these results, it seems that these 68 proteins, validated with different datasets on different platforms can be suggested as prominent candidate proteins for further investigations for biomarker detection. Moreover, based on the supportive findings, the six featured proteins can be proposed as possible biomarkers for BC, for which our laboratory has begun cell culture investigations to confirm our results. However, their efficacy in the BC subtype differentiation yet remains unclear. The global burden of BC has been previously reported [3, 4], and most developed countries in all five continents with high to moderate HDI levels suffer from BC today. Still, an increase is predicted in the next two decades, for which the countries with the highest HDI levels are still in the lead. This fact increases the urgency to uncover accurate biomarkers for early detection, prognosis assessment, and most importantly repurposing drugs or the discovery of new target agents and different treatment approaches. As a closing remark, it is of utmost importance to observe the long-term effects of the COVID-19 pandemic, which might have a reflection on the incidence and mortality rates of BC.

## Supplementary Information

**Additional file 1: Fig. S1.** Hierarchical clustering analysis of the second validation study with 52|common probes (genes) in the E-MTAB-1940 dataset. [BC and control samples are represented on the vertical axis, probes (genes) on the horizontal axis; red, black, and green colours represent high, medium, and low gene expression levels, respectively].

**Additional file 2: Table S1.** Mutual probesets in both datasets and their corresponding genes, expression levels in BC and normal tissue, fold-change and t-test *p*-values.

## Data Availability

The datasets analyzed during the current study are available in the [GEO DATASETS, NCBI] repository, [https://www.ncbi.nlm.nih.gov/gds/] with GSE13507, GSE37817 and GSE5.

## Abbreviations

BC: Bladder cancer
HDI: Human Developmental Index
GEO: Gene expression omnibus
HOPP: Heuristic Online Phenotype Prediction
GO: Gene ontology
STRING: Search Tool for the Retrieval of Interaction Genes 10.5 (STRING)
PPI: Protein-protein interaction
DEG: Differentially expressed gene
CDC2: Cell division cycle
BUB1B: BUB1 mitotic checkpoint serine/threonine kinase beta
CKAP2L: Cytoskeleton-associated protein 2-like
AURKB: Aurora kinase B
APIP: APAF-1 interacting protein
Apaf-1: Apoptotic protease activating factor-1
LGALS3: Galectin 3
