# Supplementary figures and images for: Six potential biomarkers for bladder cancer: key proteins in cell-cycle division and apoptosis pathways
Source: J Egypt Natl Canc Inst. 2022 Dec 19;34:54. doi: 10.1186/s43046-022-00153-0 (PMC9760318; doi:10.1186/s43046-022-00153-0)

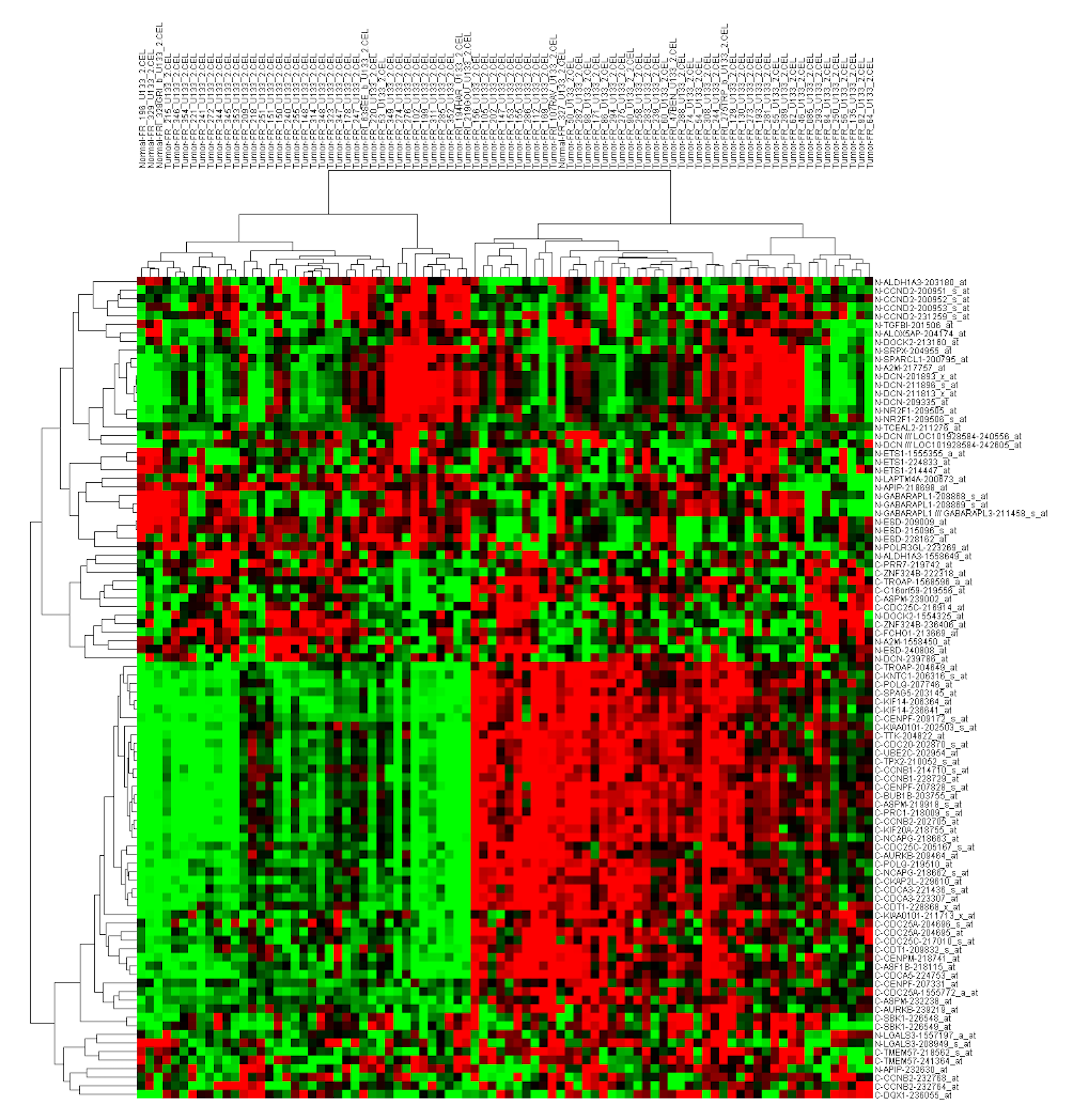

Supplement: Supplementary file 1 — Additional file 1: Fig. S1. Hierarchical clustering analysis of the second validation study with 52|common probes (genes) in the E-MTAB-1940 dataset. [BC and control samples are represented on the vertical axis, probes (genes) on the horizontal axis; red, black, and green colours represent high, medium, and low gene expression levels, respectively]. [file 43046_2022_153_MOESM1_ESM.png]
