# Supplementary material for: Six potential biomarkers for bladder cancer: key proteins in cell-cycle division and apoptosis pathways
Source: J Egypt Natl Canc Inst. 2022 Dec 19;34:54. doi: 10.1186/s43046-022-00153-0 (PMC9760318; doi:10.1186/s43046-022-00153-0)
Supplement: Supplementary file 2 — Additional file 2: Table S1. Mutual probesets in both datasets and their corresponding genes, expression levels in BC and normal tissue, fold-change and t-test p-values. [file 43046_2022_153_MOESM2_ESM.docx]

**Table S1:** Mutual probesets in both datasets and their corresponding genes, expression levels in BC and normal tissue, fold-change ​​and t-test p-values.

|  | **Common Probes** |  |  | **GSE13507** | | **GSE37817** | |
| --- | --- | --- | --- | --- | --- | --- | --- |
|  | **Illumina ID** | **Gene Symbol** | **Tumor expression** | **Fold Change** | **T-Test p-values** | **Fold Change** | **T-Test p-values** |
| 1 | ILMN_1703906 | DKFZp762E1312 | High | 1,22 | 9,6E-37 | 1,21 | 2,9E-07 |
| 2 | ILMN_1792494 | TPX2 | High | 1,27 | 3,5E-34 | 1,27 | 1,4E-06 |
| 3 | ILMN_1768291 | SPAG5 | High | 1,21 | 1,2E-33 | 1,20 | 5,3E-07 |
| 4 | ILMN_1742958 | UBE2C | High | 1,31 | 5,7E-33 | 1,32 | 5,0E-07 |
| 5 | ILMN_1702197 | C9orf140 | High | 1,21 | 3,9E-31 | 1,20 | 3,0E-06 |
| 6 | ILMN_1751776 | CKAP2L | High | 1,21 | 7,7E-29 | 1,22 | 5,3E-06 |
| 7 | ILMN_1684217 | AURKB | High | 1,25 | 1,7E-28 | 1,24 | 2,8E-07 |
| 8 | ILMN_1808347 | CDC45L | High | 1,17 | 2,2E-26 | 1,16 | 2,1E-06 |
| 9 | ILMN_1712803 | CCNB1 | High | 1,16 | 1,8E-25 | 1,17 | 5,0E-06 |
| 10 | ILMN_1749829 | DLG7 | High | 1,20 | 6,3E-24 | 1,22 | 4,8E-06 |
| 11 | ILMN_1728934 | PRC1 | High | 1,25 | 7,2E-24 | 1,26 | 8,6E-07 |
| 12 | ILMN_1808071 | KIF14 | High | 1,15 | 8,0E-24 | 1,13 | 3,9E-06 |
| 13 | ILMN_1683450 | CDCA5 | High | 1,28 | 1,7E-23 | 1,27 | 1,4E-06 |
| 14 | ILMN_1733950 | BUB1B | High | 1,21 | 1,7E-23 | 1,21 | 1,2E-06 |
| 15 | ILMN_1732516 | KNTC1 | High | 1,14 | 3,1E-23 | 1,14 | 3,2E-07 |
| 16 | ILMN_1725260 | CDC25C | High | 1,12 | 5,3E-23 | 1,11 | 2,4E-06 |
| 17 | ILMN_1651237 | CDT1 | High | 1,27 | 5,4E-23 | 1,23 | 1,1E-06 |
| 18 | ILMN_1664516 | CENPF | High | 1,25 | 7,9E-23 | 1,22 | 2,0E-06 |
| 19 | ILMN_1668814 | CENPM | High | 1,20 | 2,5E-22 | 1,22 | 4,4E-06 |
| 20 | ILMN_1654571 | FCHO1 | High | 1,13 | 3,6E-22 | 1,16 | 9,8E-07 |
| 21 | ILMN_1801939 | CCNB2 | High | 1,30 | 4,5E-22 | 1,31 | 8,8E-07 |
| 22 | ILMN_1733396 | CDC25A | High | 1,14 | 7,1E-22 | 1,14 | 3,8E-06 |
| 23 | ILMN_1708261 | SBK1 | High | 1,21 | 1,3E-21 | 1,23 | 9,4E-07 |
| 24 | ILMN_1736816 | C13orf3 | High | 1,14 | 4,0E-21 | 1,12 | 3,2E-06 |
| 25 | ILMN_1656452 | C16orf59 | High | 1,17 | 1,8E-20 | 1,17 | 7,5E-07 |
| 26 | ILMN_1663390 | CDC20 | High | 1,40 | 1,9E-20 | 1,39 | 2,0E-08 |
| 27 | ILMN_1751444 | NCAPG | High | 1,26 | 6,6E-20 | 1,28 | 5,4E-07 |
| 28 | ILMN_1815184 | ASPM | High | 1,28 | 7,7E-20 | 1,30 | 6,3E-07 |
| 29 | ILMN_1663332 | KIAA0101 | High | 1,20 | 1,6E-19 | 1,20 | 2,5E-06 |
| 30 | ILMN_1788166 | TTK | High | 1,29 | 6,3E-19 | 1,29 | 2,2E-07 |
| 31 | ILMN_1700337 | TROAP | High | 1,23 | 1,8E-18 | 1,20 | 1,1E-06 |
| 32 | ILMN_1695658 | KIF20A | High | 1,28 | 2,1E-18 | 1,29 | 3,9E-07 |
| 33 | ILMN_1770502 | DQX1 | High | 1,15 | 3,3E-18 | 1,16 | 5,1E-06 |
| 34 | ILMN_1737728 | CDCA3 | High | 1,20 | 6,3E-18 | 1,20 | 4,7E-06 |
| 35 | ILMN_1785756 | LOC729063 | High | 1,13 | 7,1E-18 | 1,12 | 2,0E-07 |
| 36 | ILMN_1695414 | ASF1B | High | 1,25 | 9,2E-18 | 1,25 | 2,2E-06 |
| 37 | ILMN_1681221 | C9orf100 | High | 1,07 | 1,5E-17 | 1,07 | 6,6E-07 |
| 38 | ILMN_1700766 | ZNF324B | High | 1,06 | 3,6E-17 | 1,05 | 1,2E-06 |
| 39 | ILMN_1718831 | TMEM57 | High | 1,06 | 4,7E-17 | 1,07 | 2,9E-07 |
| 40 | ILMN_1677509 | PRR7 | High | 1,12 | 1,3E-16 | 1,13 | 2,2E-06 |
| 41 | ILMN_1664154 | LOC440030 | High | 1,26 | 2,0E-16 | 1,29 | 2,7E-06 |
| 42 | ILMN_1740291 | POLQ | High | 1,23 | 2,6E-16 | 1,23 | 4,5E-07 |
| 43 | ILMN_1745594 | LOC401206 | Low | 0,95 | 3,2E-21 | 0,97 | 2,4E-05 |
| 44 | ILMN_1663866 | TGFBI | Low | 0,88 | 7,5E-18 | 0,87 | 4,3E-07 |
| 45 | ILMN_1709486 | SRPX | Low | 0,69 | 6,6E-16 | 0,71 | 1,6E-09 |
| 46 | ILMN_1797875 | ALOX5AP | Low | 0,82 | 4,6E-15 | 0,78 | 1,6E-09 |
| 47 | ILMN_1807439 | ALDH1A3 | Low | 0,84 | 4,9E-15 | 0,83 | 3,9E-07 |
| 48 | ILMN_1793598 | APIP | Low | 0,91 | 6,6E-15 | 0,92 | 1,3E-05 |
| 49 | ILMN_1747118 | LGALS3 | Low | 0,85 | 2,1E-14 | 0,86 | 2,5E-05 |
| 50 | ILMN_1788347 | KIAA1737 | Low | 0,90 | 2,1E-14 | 0,91 | 6,8E-06 |
| 51 | ILMN_1750347 | LOC401152 | Low | 0,87 | 2,8E-12 | 0,91 | 7,9E-06 |
| 52 | ILMN_1720285 | ESD | Low | 0,90 | 1,1E-11 | 0,91 | 5,5E-06 |
| 53 | ILMN_1799725 | DOCK2 | Low | 0,85 | 1,3E-11 | 0,82 | 4,8E-09 |
| 54 | ILMN_1760667 | POLR3GL | Low | 0,93 | 1,6E-11 | 0,93 | 4,0E-06 |
| 55 | ILMN_1745607 | A2M | Low | 0,85 | 2,0E-11 | 0,88 | 6,1E-07 |
| 56 | ILMN_1713833 | PECI | Low | 0,90 | 5,0E-11 | 0,92 | 1,1E-05 |
| 57 | ILMN_1667081 | CCND2 | Low | 0,80 | 6,2E-11 | 0,79 | 3,1E-07 |
| 58 | ILMN_1712574 | LOC647908 | Low | 0,88 | 9,1E-11 | 0,91 | 4,0E-06 |
| 59 | ILMN_1692249 | LOC641750 | Low | 0,82 | 1,0E-10 | 0,86 | 2,8E-05 |
| 60 | ILMN_1687538 | ETS1 | Low | 0,92 | 1,9E-10 | 0,93 | 1,5E-05 |
| 61 | ILMN_1671260 | GPR177 | Low | 0,87 | 2,9E-10 | 0,88 | 7,6E-07 |
| 62 | ILMN_1795251 | SPARCL1 | Low | 0,77 | 3,3E-10 | 0,80 | 2,6E-06 |
| 63 | ILMN_1667846 | GABARAPL1 | Low | 0,91 | 8,0E-10 | 0,90 | 8,1E-07 |
| 64 | ILMN_1765310 | TCEAL2 | Low | 0,73 | 8,2E-10 | 0,71 | 1,0E-08 |
| 65 | ILMN_1786197 | NR2F1 | Low | 0,79 | 9,5E-10 | 0,80 | 3,5E-06 |
| 66 | ILMN_1701748 | DCN | Low | 0,79 | 1,5E-09 | 0,80 | 6,2E-07 |
| 67 | ILMN_1695959 | C21orf63 | Low | 0,84 | 3,0E-09 | 0,81 | 1,9E-06 |
| 68 | ILMN_1745110 | LAPTM4A | Low | 0,93 | 3,9E-09 | 0,94 | 1,9E-05 |
